# The association of neutrophil-lymphocyte ratio and prognostic nutritional index with the development to chronic critical illness and their prognosis

**DOI:** 10.3389/fnut.2025.1505404

**Published:** 2025-07-23

**Authors:** Xinghua Chen, Ziwei Li, Xing Wei, Li Yao

**Affiliations:** ^1^Intensive Care Unit, The Second People’s Hospital of Hefei, Hefei Hospital Affiliated to Anhui Medical University, Hefei, Anhui, China; ^2^The Fifth Clinical School of Medicine, Anhui Medical University, Hefei, Anhui, China; ^3^Department of Cardiology, The Second People's Hospital of Hefei, Hefei Hospital Affiliated to Anhui Medical University, Hefei, Anhui, China

**Keywords:** intensive care unit, chronic critical care, neutrophil-lymphocyte ratio, Prognostic Nutrition Index, MIMIC-IV database

## Abstract

**Background:**

The association between neutrophil-lymphocyte ratio (NLR) prognostic nutritional index (PNI) and chronic critical illness (CCI) is unclear. We aimed to explore the association between NLR and PNI with CCI and whether it could be used as a tool for risk stratification in such patients.

**Methods:**

A retrospective cohort study was conducted using the Medical Information Mart for Intensive Care-IV (MIMIC-IV) database. The data collection spanned from 2008 to 2019, and the database was sourced from Beth Israel Deaconess Medical Center in Boston. The clinical data of patients who were admitted to ICU for the first time for more than 24 h were collected, including the values of NLR and PNI on the first day of admission. The primary outcomes of the study were whether critically ill patients progressed to CCI and the in-hospital all-cause mortality rate of patients with CCI. Multivariate logistic regression models were used to analyze the relationship between NLR and PNI and outcomes, and three models were used to adjust for possible confounders. Receiver operating characteristic (ROC) curves and the area under the curve (AUC) were utilized to evaluate the predictive value of these research indicators for the outcomes. Subgroup analyses were also performed to explore whether the association of the study metrics with outcome was robust across different patient populations.

**Results:**

A total of 5,637 critically ill patients were ultimately enrolled in the study, and 675 (12%) progressed to CCI, with in-hospital death occurring in 115 (17%) of these patients. In the adjusted model of critically ill patients progressing to CCI, the ORs for NLR and PNI were 1.050 (1.041–1.060) and 0.958 (0.944–0.971), respectively. The AUC were 0.755 (0.735–0.775) and 0.718 (0.697–0.739). In the adjusted model for in-hospital deaths in CCI patients, the ORs for NLR and PNI were 1.014 (1.003–1.025) and 0.951 (0.923–0.979), respectively. The AUC were 0.670 (0.619–0.721) and 0.677 (0.626–0.729), respectively. Results remain robust across patient populations.

**Conclusion:**

High NLR and low PNI levels are associated with progression to CCI and in-hospital death in critically ill patients and can be used as a valid predictive tool for poor prognosis in critically ill patients.

## Highlights

- Current high prevalence of CCI but limited research.
- NLR and PNI are associated with progression to CCI in critically ill patients.
- Both can be used as tools for early identification and evaluation of CCI patients.

## 1 Introduction

With the continuous advancement of intensive care medicine and the improvement of treatment methods for patients with acute diseases, the mortality rate of critically ill patients in intensive care units (ICU) has been greatly reduced. Nonetheless, many patients survive the acute phase but continue to require prolonged intensive care and advanced life support, a condition that is often referred to as chronic critical illness (CCI) (1). CCI is often viewed as a subacute disease condition with distinguishing features that include the need for prolonged hospitalization, significant suffering, high mortality, and significant resource consumption (2). In recent decades, the prevalence of CCI has been rising worldwide. In the United States, there are 34.4 cases of CCI per 100,000 population, with an increase of 25.76% between 2004 and 2009 (3). A multicenter cross-sectional study in China showed that 30.7% of ICU patients progressed to CCI and were often associated with severe complications, as well as respiratory and renal failure, which severely affected patient prognosis (4). A large epidemiological survey in Japan showed that the prevalence of CCI was 42.0 cases per 100,000 people, with an in-hospital mortality rate of 28.6, and 40% of patients died within 6 months (5). Given the current high morbidity and mortality characteristics of CCI, early detection of risk warning is very important.

The pathogenesis of CCI is complex. Previous studies have shown that a persistent state of inflammation and immunosuppression is closely related to the development of CCI (6). Patients with CCI are in a chronic state of inflammation due to persistent infections, repeated tissue damage and repair processes, metabolic disorders, and autoimmune responses (7). This persistent inflammatory state not only aggravates the progression of the original disease but also inhibits the body’s normal immune response (8). In addition, critically ill patients are more likely to be malnourished because they are often accompanied by elevated basal metabolic rates, inadequate medical feeding, and intestinal dysfunction (9). Studies have found that up to 38–78% of ICU patients have malnutrition, which is closely related to increased mortality, prolonged hospital stay, and prolonged mechanical ventilation (10).

Neutrophil-lymphocyte ratio (NLR) is an affordable and practical hematologic measure of a patient’s inflammatory and immune status (11). Many previous studies have shown that NLR is associated with poor prognosis in many acute critical illnesses such as sepsis, trauma, stroke, and cardiovascular accidents (12–15). In addition, NLR has been associated with many chronic diseases (e.g., cardiovascular disease, diabetes, cancer, etc.) and is recognized as a new indicator of chronic inflammation (16–18). The Prognostic Nutrition Index (PNI) was originally proposed by Buzby et al. (19). And was initially used to assess Immune nutritional status in patients undergoing gastrointestinal surgery (20–22). It was later shown to have a significant association with the prognosis of patients with various cancers. In addition, it has been found that the PNI shows good predictive efficacy in sepsis, heart failure, acute myocardial infarction, and, more recently, in novel coronavirus pneumonia (23–26).

However, few studies have been conducted to explore targeted early warning approaches for the current high incidence of CCI. Considering the possibility of abnormal immune-inflammatory response and nutritional status of CCI patients. The aim of this study was to evaluate the association of NLR and PNI with progression to CCI in critically ill patients and poor prognosis in patients with CCI and to investigate whether both could be used for risk stratification of ICU patients.

## 2 Materials and methods

### 2.1 Research source

The Medical Information Mart for Intensive Care-IV database [MIMIC-IV, version 2.2; (27)] is an open-access intensive care medicine database developed by the Massachusetts Institute of Technology in collaboration with Harvard Medical School. The database collects clinical data from 2008 through 2019 on more than 50,000 ICU patients from Beth Israel Deaconess Medical Center in Boston. Data sources for the database include a variety of medical devices and systems such as electronic medical record systems, vital sign monitors, and laboratory information systems. It provides a wide range of data including basic information, physiological parameters, laboratory test results, medication information, treatment process, and disease diagnosis. In addition the MIMIC-IV database provides follow-up data for 1 year after the patient’s last ICU discharge, enabling researchers to conduct long-term studies of the disease process and treatment effects. Patient information has been treated confidentially and there are no ethical related issues. Our researcher, Xinghua Chen, completed the National Institutes of Health online training program and was approved for access to raw data (certification number: 58951192).

### 2.2 Subjects of the study

We used the consensus definition developed by the Research Triangle Institute to define CCI (28). The CCI group was defined as an ICU stay of at least 8 days and at least one of the following conditions was met during the hospitalization: prolonged acute mechanical ventilation (a single episode of mechanical ventilation of at least 96 h); tracheotomy; sepsis; severe wounds; stroke (including ischemic stroke and hemorrhagic stroke) and traumatic brain injury. The non-ICU group was defined as an ICU stay shorter than 8 days without death or abandonment of treatment within 8 days.

### 2.3 Inclusion and exclusion

**Figure 1:**
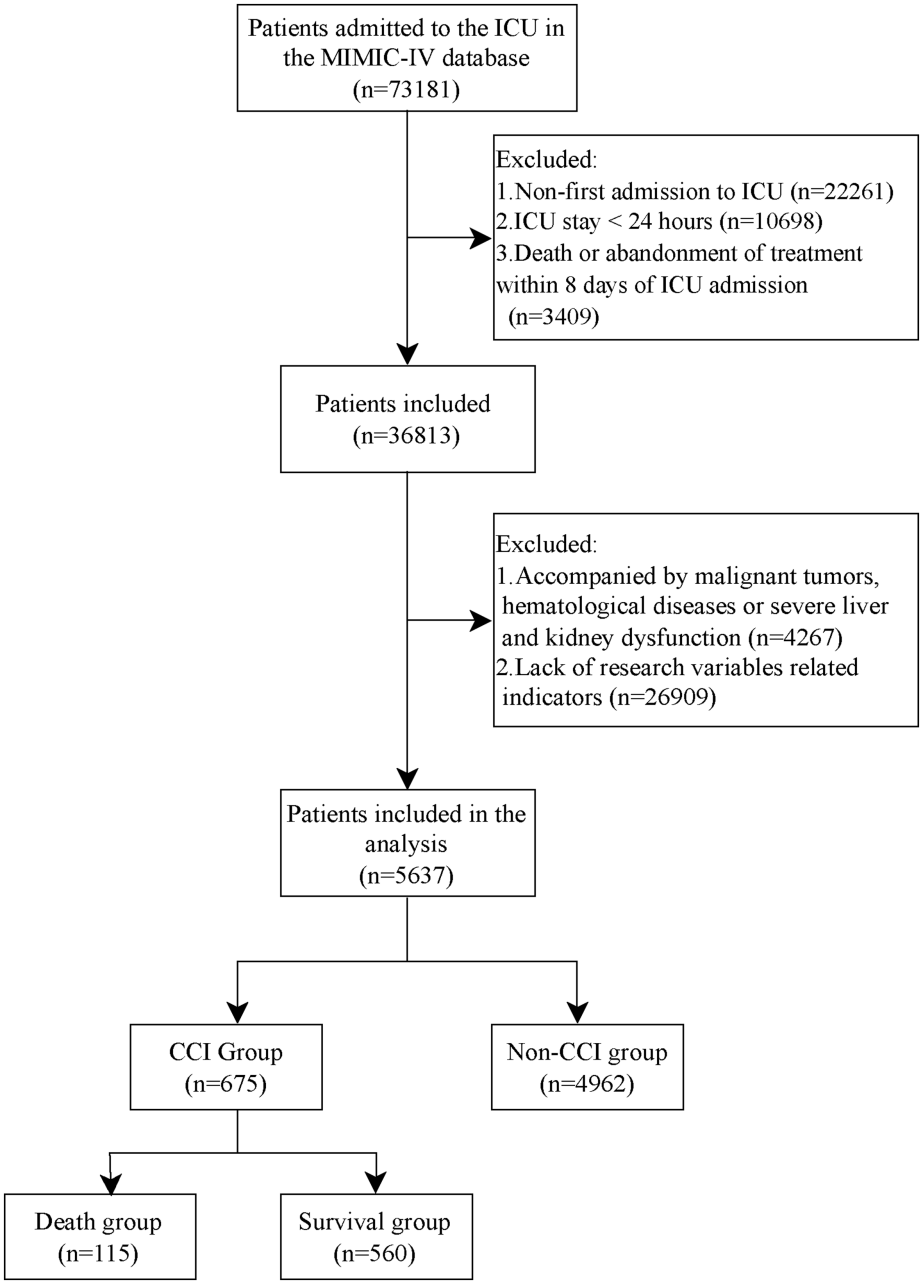
Flow chart.

Inclusion criteria: (a) first admission to the ICU; (b) ICU stay greater than 24 h; Exclusion criteria: (a) death or abandonment of treatment within 8 days after ICU admission; (b) with malignant tumors, hematological disorders, immunodeficiency disorders, or severe hepatic or renal impairment; (c) lack of indexes related to the study variables. Figure 1 flow chart.

### 2.4 Data collection

Patient data were collected in 5 main areas. These included (1) demographic data: age, sex, body mass index (BMI); (2) comorbidities: diabetes mellitus (DM), coronary artery disease (CAD), COPD, acute heart failure (AHF), and pneumonia; (3) severity of illness scores: Acute Physiology Score-III (APS-III), Oxford Acute Severity of Illness Score (OASIS), Sequential Organ Failure Assessment (SOFA) score, Glasgow Coma Scale (GCS) score; (4) Laboratory tests within 24 h of admission to ICU: red blood cell count (RBC), white blood cell count (WBC), platelet count (PLT), neutrophil count, lymphocyte count, monocyte count, hemoglobin (Hb), albumin (Alb), serum creatinine (SCr), NLR, PNI; (5) Treatment received within 24 h of admission to ICU: use of vasoactive drugs, use of sedative drugs, nasal nutrition, parenteral nutrition (PN). Information on patient comorbidities was collected according to the International Classification of Diseases (ICD-9 and ICD-10). None of the study variables were missing, and the remaining variables were controlled within 20%.

### 2.5 Variables and outcomes

NLR calculation equation: NLR = N/L. PNI calculation equation: PNI = L × 5 + Alb. N, L, and Alb represent peripheral neutrophil count (×10^9^/L), lymphocyte count (×10^9^/L), and serum albumin (g/L) respectively. The primary outcomes were whether the critically ill patients progressed to CCI and in-hospital all-cause mortality in the CCI group. Secondary outcomes were length of hospital stay, length of ICU stay, and 60-day, 180-day, and 1-year all-cause mortality in the CCI group.

### 2.6 Statistical analysis

We used Navicat Premium 16.0 software to extract medical records from the database, and STATA 15.1 and R 4.3.1 software for data collation and analysis. For variables with a missing amount of less than 20%, the “MICE” package of R software is used for multiple interpolation filling. For normally distributed continuous variables, we presented data as mean (standard deviation), utilizing t-tests for intergroup comparisons. Skewed continuous variables were represented by median (interquartile range), with the Mann–Whitney U test facilitating comparisons between groups. In the case of categorical variables, we depicted them using frequency (proportion), employing chi-square tests to discern differences across groups.

Univariate and multivariate logistic regression models were used to assess the effects of NLR and PNI on progression to CCI in critically ill patients and in-hospital all-cause mortality in the CCI group. According to the factors that may affect the research variables and outcomes. We used three models to adjust for potential confounders. Model 1: adjusted for demographic parameters (age, sex, BMI); Model 2: Model 1 plus comorbidities and various scores (DM, COPD, pneumonia, APS-III, and SOFA score); and Model 3: Model 2 plus laboratory tests and treatments received (Hb, SCr, use of vasoactive drugs, use of sedative drugs, and nasal nutrition). Plotting scatter plots of study metrics versus APS-III and SOFA score, and performing Spearman analysis to assess the association of study indicators with disease severity. The association between study variables and outcomes was further assessed using restricted cubic spline (RCS) curves.

By drawing the receiver operator characteristic (ROC) curve and comparing the area under the ROC curve (AUC), the predictive value of the research indicators for the endpoints was analyzed. Incorporate study indicators into existing disease severity scores to compare whether study indicators improve the accuracy of commonly utilized scores in predicting study outcomes. Using the Delong test to compare prediction performance.

Descriptive statistics were performed on the prognostic data of patients in the CCI group. Kaplan–Meier analysis was used to assess the survival of patients in the CCI group at different levels of study variables, and differences between groups were assessed by the log-rank test.

In addition, to further assess whether there were differences in the impact of the study variables in different populations. We performed subgroup analyses of patients with different ages, sexes, SOFA scores, and with or without concomitant DM, COPD, and pneumonia, as well as with or without the use of vasoactive drugs and nasal nutrition. Subgroup analyses were conducted in the framework of multivariate logistic regression model 3. *p* < 0.05 was considered a statistically significant difference.

## 3 Results

### 3.1 Baseline data analysis

There were 50,920 first-time ICU admissions in the MIMIC-IV database, of which a total of 3,661 (7.19%) met the definition of CCI. The distribution of clinical factors in the definition of patients with CCI is shown in Supplementary Table S1. Based on the inclusion and exclusion criteria, 5,637 patients were finally included in the study, including 675 in the CCI group and 4,962 in the non-CCI group. The baseline information of the patients in both groups is shown in Table 1. The results showed that the differences in BMI, CAD, COPD, AHF, pneumonia, APS-III, OASIS, SOFA score, GCS score, WBC count, neutrophil count, lymphocyte count, monocyte count, Alb, SCr, NLR, PNI, use of vasoactive drugs, use of sedative drugs, nasal nutrition, and PN between two groups were statistically significant (*p* < 0.05).

**Table 1:** Baseline characteristics of patients.

Variables	Non-CCI group (*n* = 4,962)	CCI group (*n* = 675)	*p*	Survival group (*n* = 560)	Death group (*n* = 115)	*p*
Demographic						
Age, years	67.6 (54.7–78.5)	66.3 (55.2–76.9)	0.118	65.1 (54.1–76.3)	70.1 (59.6–78.8)	0.006
Sex, male	2,736 (55.1%)	365 (54.1%)	0.631	296 (52.9%)	69 (60%)	0.195
BMI, kg/m^2^	27.1 (23.3–31.8)	28.1 (23.5–34.0)	<0.001	28.1 (23.5–33.2)	28.2 (24.2–36.5)	0.328
Comorbidity						
DM	1,471 (29.6%)	183 (27.1%)	0.190	145 (25.9%)	38 (33%)	0.145
CAD	1,343 (27.1%)	129 (19.1%)	<0.001	98 (17.5%)	31 (27%)	0.026
COPD	549 (11.1%)	101 (15%)	0.004	80 (14.3%)	21 (18.3%)	0.345
AHF	670 (13.5%)	119 (17.6%)	0.005	97 (17.3%)	22 (19.1%)	0.742
Pneumonia	978 (19.7%)	459 (68%)	<0.001	373 (66.6%)	86 (74.8%)	0.109
Various scores						
APS-III	39.0 (29.0–52.0)	69.0 (50.0–92.0)	<0.001	66.0 (47.0–87.5)	90.0 (64.5–108.5)	<0.001
OASIS	30.0 (25.0–36.0)	41.0 (35.0–48.0)	<0.001	40.0 (35.0–47.0)	44.0 (37.0–50.5)	0.002
SOFA score	4.0 (2.0–6.0)	9.0 (5.0–12.0)	<0.001	8.0 (5.0–12.0)	11.0 (8.0–13.0)	<0.001
GCS score	14.0 (13.0–15.0)	9.0 (6.0–12.0)	<0.001	9.0 (6.0–12.0)	6.0 (3.0–10.0)	<0.001
Laboratory tests						
RBC count, ×10^9^/L	3.6 (3.1–4.1)	3.6 (3.1–4.2)	0.482	3.6 (3.2–4.2)	3.6 (3.0–4.1)	0.193
WBC count, ×10^9^/L	10.5 (7.8–14.2)	12.2 (9.0–16.5)	<0.001	12.1 (8.8–16.1)	13.4 (9.6–17.9)	0.078
PLT count, ×10^9^/L	183.0 (138.0–240.0)	178.0 (129.0–239.0)	0.087	180.5 (131.0–240.5)	161.0 (122.5–235.0)	0.130
Neutrophil count, ×10^9^/L	8.7 (5.8–12.7)	10.3 (6.7–15.0)	<0.001	9.7 (6.4–14.5)	12.8 (10.2–18.8)	<0.001
Lymphocyte count, ×10^9^/L	1.5 (1.1–2.2)	0.7 (0.4–1.4)	<0.001	0.8 (0.4–1.4)	0.6 (0.3–1.1)	0.009
Monocyte count, ×10^9^/L	0.7 (0.4–1.0)	0.7 (0.4–1.1)	0.030	0.7 (0.4–1.1)	0.7 (0.4–1.1)	0.819
Hb, g/L	108.0 (92.0–123.0)	108.0 (92.0–123.0)	0.429	109.0 (92.5–125.0)	103.0 (90.5–119.5)	0.049
Alb, g/L	37.0 (31.0–41.0)	32.0 (26.0–38.0)	<0.001	33.0 (27.0–39.0)	27.0 (22.5–33.5)	<0.001
SCr, g/L	90.0 (70.0–120.0)	100.0 (70.0–170.0)	<0.001	100.0 (70.0–160.0)	130.0 (95.0–205.0)	<0.001
NLR	5.5 (3.1–9.4)	13.2 (6.8–25.9)	<0.001	11.8 (6.4–22.6)	21.2 (10.7–36.8)	<0.001
PNI	44.7 (38.1–50.7)	36.4 (30.2–44.2)	<0.001	37.9 ± 9.3	32.0 ± 8.3	<0.001
Treatment received						
Use of vasoactive drugs	1,620 (32.6%)	450 (66.7%)	<0.001	352 (62.9%)	98 (85.2%)	<0.001
Use of sedative drugs	2017 (40.6%)	598 (88.6%)	<0.001	488 (87.1%)	110 (95.7%)	0.014
Nasal nutrition	544 (11%)	516 (76.4%)	<0.001	428 (76.4%)	88 (76.5%)	1.000
PN	95 (1.9%)	40 (5.9%)	<0.001	30 (5.4%)	10 (8.7%)	0.244

CCI, chronic critical illness; BMI, body mass index; DM, diabetes mellitus; CAD, coronary artery disease; AHF, acute heart failure; COPD, chronic obstructive pulmonary disease; APS-III, Acute Physiology Score-III; OASIS, Oxford Acute Severity of Illness Score; SOFA, Sequential Organ Failure Assessment; GCS, Glasgow Coma Scale; RBC, red blood cell; WBC, white blood cell; PLT, platelet; Hb, hemoglobin; Alb, albumin; SCr, serum creatinine; NLR, neutrophil-lymphocytes ratio; PNI, prognosis nutrition index; PN, parenteral nutrition. Values are the mean ± standard deviation, median (interquartile range), or *n* (%).

A total of 115 (17.0%) in the CCI group died during hospitalization. Baseline information for the death and survival groups is shown in Table 1. The results showed that there were statistically significant differences between the two groups in terms of age, CAD, APS-III, OASIS, SOFA score, GCS score, neutrophil counts, lymphocyte counts, Hb, Alb, SCr, NLR, PNI, use of vasoactive drugs, and use of sedative drugs (*p* < 0.05). Violin plots showed that NLR was significantly higher in the CCI and in-hospital death groups than in the non-CCI and survival groups, whereas the levels of PNI were reversed (Supplementary Figure S1).

### 3.2 Association analysis

Multivariate logistic regression analysis showed that NLR was a risk factor for progression to CCI in critically ill patients. The results were significant in the unadjusted model (OR [95%CI], 1.067 [1.060–1.075], *p* < 0.001), model 1 (OR [95%CI], 1.068 [1.060–1.075], *p* < 0.001), model 2 (OR [95%CI], 1.055 [1.048–1.063], *p* < 0.001) and model 3 (OR [95%CI], 1.050 [1.041–1.060], *p* < 0.001). PNI is a protective factor. The results were also significant in the unadjusted model (OR [95%CI], 0.913 [0.904–0.922], *p* < 0.001), model 1 (OR [95%CI], 0.912 [0.903–0.921], *p* < 0.001), model 2 (OR [95%CI], 0.930 [0.920–0.940], *p* < 0.001) and model 3 (OR [95%CI], 0.958 [0.944–0.971], *p* < 0.001). In the CCI cohort, we found similar associations between NLR and PNI and in-hospital mortality (Table 2).

**Table 2:** Multivariate logistic regression analysis of progress to CCI in critically ill patients and in-hospital mortality of patients in the CCI group.

Outcomes	NLR		PNI	
	OR (95%CI)	*p*	OR (95%CI)	*p*
Progress to CCI in critically ill patients				
Unadjusted model	1.067 (1.060–1.075)	<0.001	0.913 (0.904–0.922)	<0.001
Model 1	1.068 (1.060–1.075)	<0.001	0.912 (0.903–0.921)	<0.001
Model 2	1.036 (1.029–1.043)	<0.001	0.973 (0.962–0.984)	<0.001
Model 3	1.050 (1.041–1.060)	<0.001	0.958 (0.944–0.971)	<0.001
In-hospital mortality of patients in the CCI group				
Unadjusted model	1.020 (1.011, 1.029)	<0.001	0.929 (0.907, 0.952)	<0.001
Model 1	1.018 (1.009, 1.027)	<0.001	0.928 (0.905, 0.951)	<0.001
Model 2	1.013 (1.003, 1.022)	0.009	0.948 (0.921, 0.974)	<0.001
Model 3	1.014 (1.003, 1.025)	0.013	0.951 (0.923, 0.979)	0.001

Model 1: After adjusting for age, sex, and BMI; Model 2: adjusted for model 1, additionally adjusted for DM, COPD, pneumonia, APS-III, and SOFA score; Model 3: adjusted for model 2, additionally adjusted for Hb, SCr, use of vasoactive drugs, use of sedative drugs, nasal nutrition.

**Figure 2:**
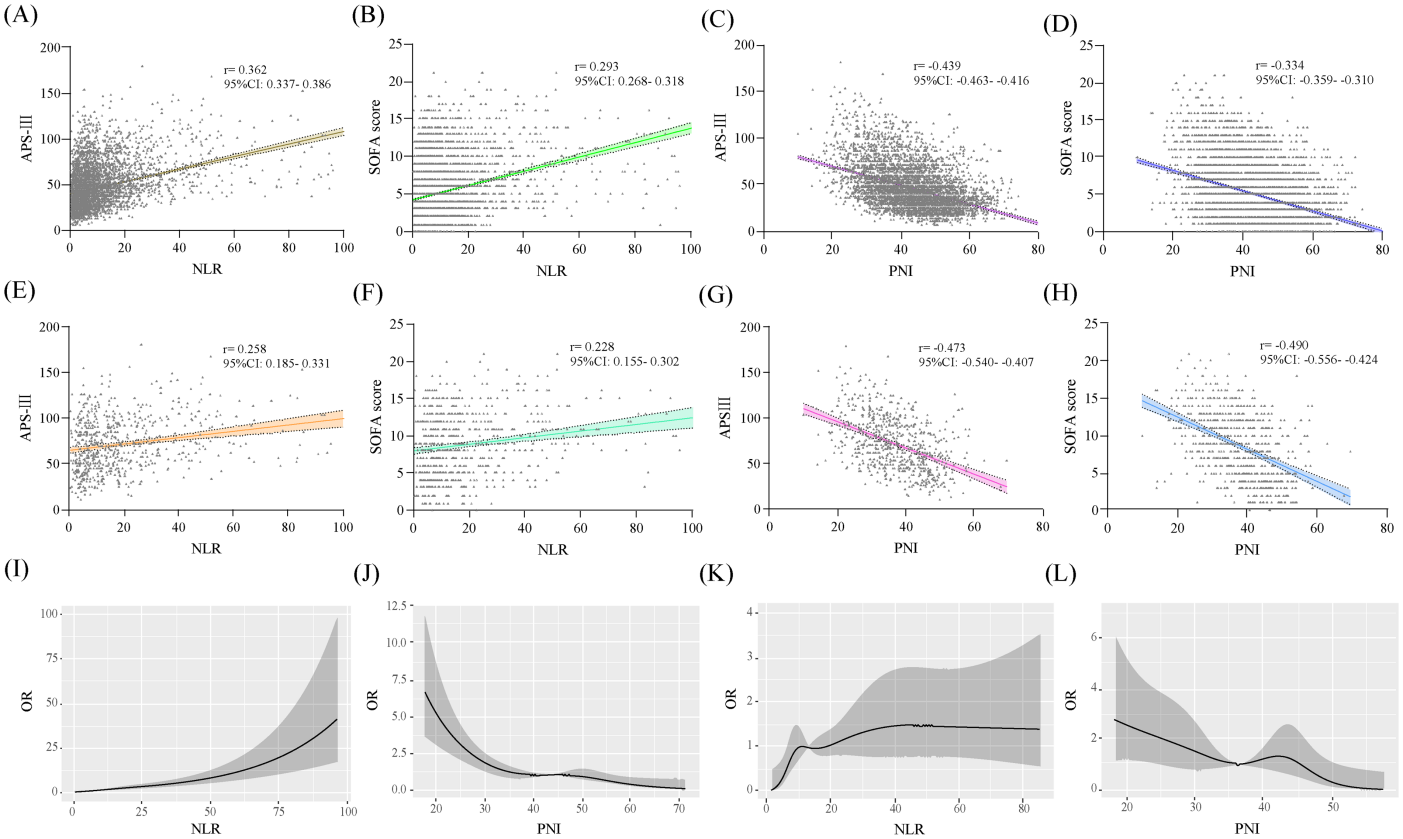
Scatterplots and RCS curves of the study variables. Scatterplots of study variables **(A–H)**. **A–D** are in the total study population and **E–H** are in the CCI group. RCS curves of the study variables: **I,J** are the associations of the study variables with progression to CCI, and **K,L** are the associations of the study variables with in-hospital death in patients with CCI.

Spearman’s analysis showed that NLR was significantly positively correlated with APS-III and SOFA scores, while PNI was significantly negatively correlated in the total study population. And these associations are also significant in the CCI cohort (Figures 2A–H). Within the framework of multifactorial logistic regression model 3, RCS curves were plotted to further assess the association between NLR and PNI levels on study outcomes. The results showed that with increasing levels of NLR, the higher the risk of patients progressing to CCI and CCI patients experiencing in-hospital death. The opposite was true for PNI (Figures 2I–L).

### 3.3 Predictive effects analysis

**Figure 3:**
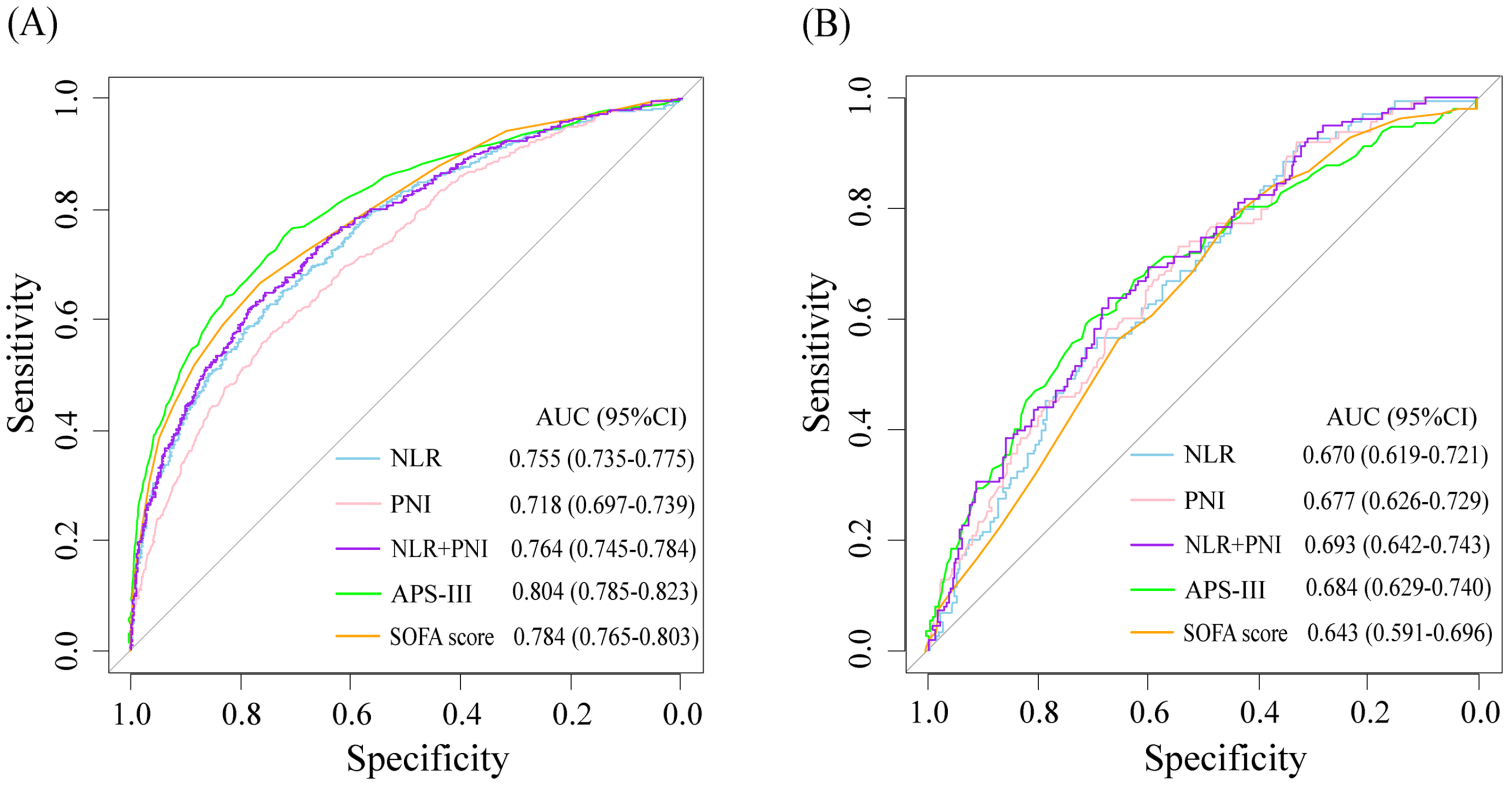
ROC curves for study variables and disease severity scores were used to predict progression to CCI in critically ill patients **(A)** and in-hospital mortality in patients with CCI **(B)**.

NLR and PNI showed certain predictive value for the progression of critically ill patients to CCI. The AUC were 0.755 (95%CI: 0.735–0.775) and 0.718 (95%CI: 0.697–0.739), respectively. When the two variables were combined as predictors, the AUC was 0.764 (95%CI: 0.745–0.784). Moreover, when combined with other common disease severity scores as predictors, the predictive accuracy of APS-III (AUC: 0.804 vs. 0.829, DeLong *p* < 0.001) and SOFA score (AUC: 0.784 vs. 0.820, DeLong *p* < 0.001) was improved. In the CCI cohort, NLR and PNI also showed certain predictive value for in-hospital mortality in patients, and could also improve the predictive value of each score (Table 3 and Figure 3).

**Table 3:** Predictive performance of each model for different outcomes.

Models	AUC (95%CI)	Models	AUC (95%CI)	*p* for comparison
Progress to CCI				
NLR	0.755 (0.735–0.775)			
PNI	0.718 (0.697–0.739)			
NLR + PNI	0.764 (0.745–0.784)			
APSIII	0.804 (0.785–0.823)	+NLR + PNI	0.829 (0.812–0.847)	<0.001
SOFA score	0.784 (0.765–0.803)	+NLR + PNI	0.820 (0.802–0.838)	<0.001
In-hospital mortality				
NLR	0.670 (0.619–0.721)			
PNI	0.677 (0.626–0.729)			
NLR + PNI	0.693 (0.642–0.743)			
APSIII	0.684 (0.629–0.740)	+NLR + PNI	0.720 (0.671–0.769)	0.032
SOFA score	0.643 (0.591–0.696)	+NLR + PNI	0.701 (0.651–0.750)	0.008

AUC, area under the ROC curve; other abbreviations are as same as Table 1.

### 3.4 Prognostic analysis

**Figure 4:**
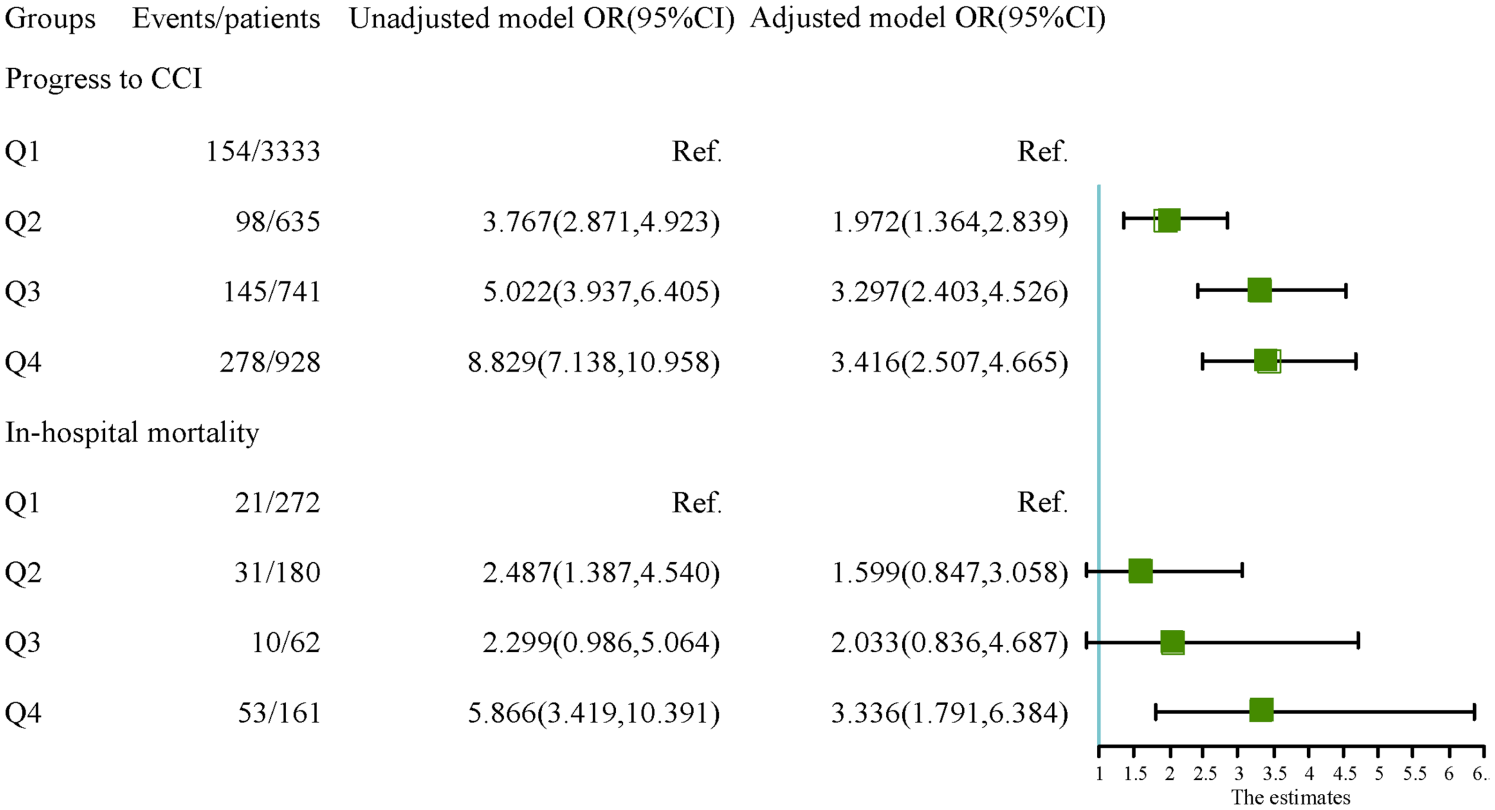
The relationship of each group with the outcomes.

According to the maximum value of the Yoden index in the ROC curve, the optimal cut-off point of the research variables is determined. In the study population, the cutoff values of NLR and PNI in the ROC curve were 9.345 and 37.825, respectively. According to the optimal cut-off value of the study variables, the population was divided into four groups: Q1 group (NLR < 9.35, PNI > 37.83), Q2 group (NLR < 9.35, PNI < 37.83), Q3 group (NLR > 9.35, PNI > 37.83) and Q4 group (NLR > 9.35, PNI < 37.83). In the cohort of patients in the CCI group, the cutoff values of NLR and PNI in the ROC curve were 19.50 and 36.48, respectively, and the patients were also divided into four groups. The Q1 group with the lowest incidence of outcome was used as the reference group. The OR values and 95% confidence intervals of each group in the unadjusted model and the fully adjusted model (Model 3) were recorded in Figure 4. The results showed that patients in groups Q2, Q3, and Q4 had a higher risk of progressing to CCI compared to group Q1. However, in the CCI cohort, the risk of in-hospital death was not statistically different between patients in groups Q2 and Q3 with high NLR levels or low PNI levels alone and those in group Q1. In contrast, the risk of in-hospital death in patients in group Q4 with combined high NLR and low PNI levels was significantly higher than that in group Q1, and the difference was statistically significant.

**Figure 5:**
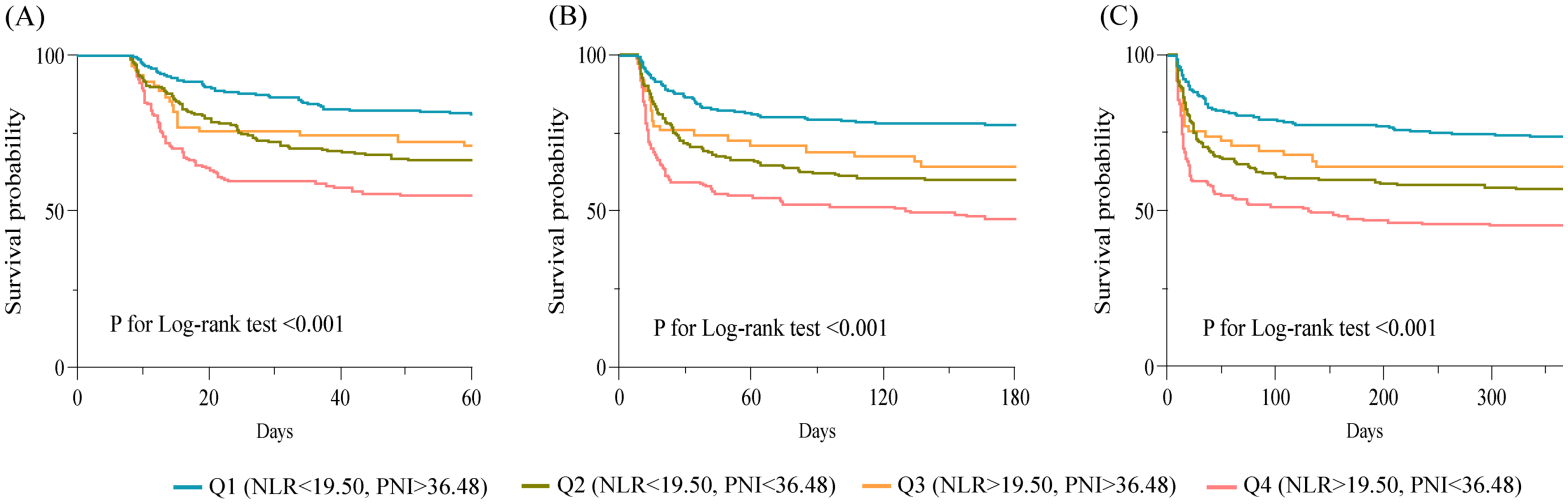
Kaplan–Meier survival curves for 60-day **(A)**, 180-day **(B)** and 1-year **(C)** survival in different groups of patients.

Descriptive statistical analysis was performed on the prognosis of the four groups of patients in the CCI cohort. The results showed that the Q1 group had lower in-hospital mortality, 60-day, 180-day, and 1-year mortality than the Q2, Q3, and Q4 groups. There was no statistically significant difference between the four groups in terms of length of ICU stay. However, in terms of length of hospitalization, patients in the Q3, and Q4 groups had shorter hospital stays, which may be due to the higher risk of in-hospital death in the Q3, and Q4 groups. After excluding those who experienced in-hospital deaths, we found that the length of the hospital stay was shorter in the Q1 group than in the Q2, Q3, and Q4 groups. However, there was still no statistically significant difference between the four groups in terms of the length of ICU stay (Table 4). The Kaplan–Meier curve of the four groups of patients is shown in Figure 5. The 60-day survival rate, 180-day survival rate, and 1-year survival rate of patients in the Q1 group were higher than those in the other three groups, and the differences between the groups were statistically significant by log-rank test (*p* < 0.05).

**Table 4:** Outcomes of patients in each group in the CCI cohort.

Outcomes	total (*n* = 675)	Q1 (*n* = 272)	Q2 (*n* = 180)	Q3 (*n* = 62)	Q4 (*n* = 161)	*p*
Hospitalization time, days	20.0 (13.9–30.6)	19.6 (13.7–29.9)	23.4 (15.9–32.0)	16.3 (12.6–29.2)	18.8 (13.2–29.2)	0.021
Length of ICU stay, days	13.0 (10.0–18.5)	13.1 (10.1–19.6)	13.2 (9.9–18.3)	13.4 (9.8–17.6)	12.5 (10.0–16.8)	0.658
In-hospital mortality, (%)	115 (17%)	21 (7.7%)	31 (17.2%)	10 (16.1%)	53 (32.9%)	<0.001
60-day mortality, (%)	201 (29.8%)	51 (18.8%)	60 (33.3%)	18 (29%)	72 (44.7%)	<0.001
180-day mortality, (%)	239 (35.4%)	61 (22.4%)	72 (40%)	22 (35.5%)	84 (52.2%)	<0.001
1-year mortality, (%)	258 (38.2%)	71 (26.1%)	77 (42.8%)	22 (35.5%)	88 (54.7%)	<0.001
Exclusion of patients with in-hospital deaths						
Hospitalization time, days	22.0 (15.8–32.7)	20.4 (14.0–30.7)	24.5 (17.0–35.6)	20.7 (13.3–31.2)	23.0 (17.2–33.5)	0.012
Length of ICU stay, days	13.2 (10.0–18.8)	13.2 (10.0–19.5)	12.9 (9.8–18.3)	13.4 (10.1–23.5)	12.6 (10.0–17.4)	0.603

Values are the median (interquartile range), or *n* (%).

### 3.5 Subgroup analysis

Subgroup analysis was performed on patients with age, sex, SOFA score, DM, COPD, pneumonia, use of vasoactive drugs, and nasal nutrition as stratification factors. The results showed that in different populations, compared with the Q1 group, the Q2, Q3, and Q4 groups had a higher risk of progression to CCI (Table 5). Interaction tests showed that the group of patients with higher SOFA scores or needing vasoactive drugs were more likely to progress to CCI when accompanied by high NLR and low PNI levels. In the CCI cohort, subgroup analysis also had similar results (Supplementary Table S2).

**Table 5:** Subgroup analysis of the relationship between groups and progression to CCI by odds ratio.

Subgroups	Groups				*p* for interaction
	Q1	Q2	Q3	Q4	
Age					0.168
<65	Ref.	1.752 (1.029–2.960)	2.806 (1.745–4.502)	2.722 (1.685–4.404)	
≥65	Ref.	2.082 (1.221–3.508)	4.060 (2.614–6.325)	4.100 (2.705–6.248)	
Sex					0.919
Female	Ref.	2.088 (1.200–3.589)	3.540 (2.201–5.698)	3.279 (2.087–5.176)	
Male	Ref.	1.913 (1.159–3.134)	3.249 (2.115–4.991)	3.658 (2.382–5.638)	
SOFA score					<0.001
<5	Ref.	0.647 (0.209–1.639)	3.965 (2.373–6.606)	2.404 (1.112–5.015)	
≥5	Ref.	2.986 (1.930–4.625)	3.676 (2.425–5.590)	4.424 (3.071–6.418)	
DM					0.353
No	Ref.	1.884 (1.212–2.910)	3.055 (2.109–4.424)	2.873 (1.978–4.181)	
Yes	Ref.	2.127 (1.054–4.204)	4.252 (2.271–8.008)	5.119 (2.893–9.175)	
COPD					0.761
No	Ref.	2.051 (1.385–3.023)	3.307 (2.348–4.654)	3.384 (2.408–4.765)	
Yes	Ref.	1.991 (0.623–6.242)	3.622 (1.479–9.237)	3.468 (1.534–8.226)	
Pneumonia					0.908
No	Ref.	1.975 (1.130–3.383)	3.584 (2.258–5.651)	3.439 (2.122–5.572)	
Yes	Ref.	2.104 (1.253–3.535)	3.534 (2.237–5.626)	3.449 (2.250–5.330)	
Use of vasoactive drugs					<0.001
No	Ref.	1.713 (0.944–3.011)	4.814 (3.139–7.396)	2.088 (1.205–3.580)	
Yes	Ref.	2.495 (1.506–4.135)	2.806 (1.716–4.597)	5.196 (3.427–7.955)	
Nasal nutrition					0.260
No	Ref.	2.064 (1.145–3.626)	3.488 (2.099–5.740)	4.621 (2.835–7.556)	
Yes	Ref.	1.812 (1.120–2.934)	3.186 (2.083–4.910)	2.757 (1.847–4.139)	

The abbreviations are as same as Table 1.

## 4 Discussion

This study is a retrospective study based on the MIMIC-IV database. According to the consensus definition developed by the Research Triangle Institute, we found that prolonged acute mechanical ventilation, sepsis, and stroke are the top three common causes of chronic critical patients. Some previous epidemiological surveys of CCI have also reached similar conclusions. In this study, we confirmed that NLR and PNI are significantly correlated with progression to CCI in critically ill patients and in-hospital death in patients with CCI, which can be used as a potential indicator for risk stratification of such patients (Table 2). Moreover, this association remained robust across patient groups. Among ICU patients, patients with high NLR and low PNI should be paid more attention (Table 5). In addition, we also found that NLR combined with PNI can be used as an effective predictive tool for poor prognosis in critically ill patients, and can also improve the predictive accuracy of common disease severity scores (Table 3).

We assessed the association between study variables and outcomes through multivariate logistic regression models. We selected specific variables to adjust the models to ensure the validity and comprehensiveness of the analysis. In Model 2, we adjusted for DM, COPD, pneumonia, APSIII, and SOFA scores. Adjustments for DM and COPD were made because these chronic diseases affect the immune-inflammatory state of patients over time. For example, type 2 diabetes promotes the release of pro-inflammatory factors and lymphocyte apoptosis, and many patients with COPD have a persistent neutrophilic infiltrate (29, 30). Pneumonia is a common acute infectious disease in the ICU and has a significant impact on the patient’s inflammatory status. The APSIII score and the SOFA score provide a comprehensive picture of the patient’s overall critical status at the time of admission to the ICU and help to exclude the confounding effect of baseline criticality on CCI outcomes. In Model 3, we adjusted for Hb, SCr, use of vasoactive and sedative medications, and nasal nutrition. Hb reflects the patient’s nutritional status, and anaemia-induced hypoxia can exacerbate inflammatory responses (31). SCr levels correlate with renal function, and fluid retention due to acute renal injury can affect albumin levels. The use of vasoactive drugs reflects circulatory status and can influence immunoinflammation, whereas sedative drugs are associated with ICU-acquired weakness (32, 33). Nasogastric nutrition was considered due to its effect on albumin levels at ICU admission.

A growing body of research suggests that the immune-inflammatory response plays a profound and sustained role in the acute morbidity and chronic recovery of critically ill patients. During the acute onset, critically ill patients experience a significant inflammatory response. During this period, the immune system is highly active, with massive proliferation of inflammatory cells (mainly granulocytes) and release of large amounts of inflammatory factors such as tumor necrosis factor-alpha and interleukins (IL-1, IL-6) to fight infection and injury (34). However, this increased bone marrow production after acute injury comes at the expense of lymphocytes and hematopoiesis (35). It has been found that in the early stages of sepsis (within 48 h), large numbers of immature neutrophils are released, and the expression of CD10 and CD16 on these immature granulocytes inhibits lymphocyte proliferation and induces apoptosis in lymphocytes (36). These immature granulocytes are also known as myeloid derived suppressor cells (MDSC). Studies have shown that persistently elevated MDSC levels in sepsis survivors are an important reason for the subsequent development of persistent immunosuppression (37, 38), which is closely associated with the progression of critically ill patients to CCI (6).

Malnutrition has been a persistent problem in ICU patients. A recent meta-analysis showed that about 15–68% of ICU patients were diagnosed with malnutrition using the Global Malnutrition Leadership Initiative criteria (39). This is related to factors such as increased metabolic stress, eating disorders, and intestinal dysfunction that are common in ICU patients. One study found that mechanically ventilated patients were more likely to be underfed with energy (OR: 2.1, 95%CI: 1.1–4.0) and protein (OR: 15.7, 95%CI: 4.9–50.8), as well as have reduced plasma albumin (OR: 2.9, 95%CI: 1.3–6.5), compared to nonventilated patients (40). Malnutrition in critically ill patients is correlated with many poor prognoses, including delayed wound healing, increased incidence of nosocomial infections, prolonged mechanical ventilation, and increased all-cause mortality (41, 42). A study of 821 ICU patients found that the higher the patient’s modified Critical Care Nutritional Risk Score at admission, the higher the survivor’s risk of post-discharge impairment in activities of daily living, and the more likely the survivor was to be admitted to the Rehabilitation Facility, Long-Term Care Center, and Nursing Facility post-discharge (33).

The predictive value of NLR and PNI for CCI progression stems from their biological plausibility in reflecting systemic inflammatory-immune-nutritional dysregulation. NLR, as a ratio of neutrophil-to-lymphocyte counts, captures the dual dynamics of innate immune activation (via neutrophils) and adaptive immune exhaustion (via lymphopenia), both of which are linked to prolonged tissue damage and impaired recovery in critically ill patients (43, 44). Similarly, PNI, calculated from serum albumin and lymphocyte counts, integrates nutritional depletion and immune incompetence—two interdependent factors exacerbating catabolic states and infection susceptibility in ICU settings. While conventional scores like APS-III and SOFA excel in quantifying acute physiological derangements and organ failure severity, they inherently ignore the persistent inflammation, immunosuppression, and catabolic states that influence the post-acute trajectory (6). Our findings demonstrate that integrating NLR and PNI with APS-III/SOFA synergistically enhances predictive accuracy, as these biomarkers provide complementary insights into patients’ intrinsic resilience beyond acute-phase severity. Clinically, this multimodal approach addresses a critical gap in risk stratification: identifying patients who, despite surviving initial physiological insults, remain vulnerable to CCI due to persistent inflammatory-metabolic exhaustion. Such integration aligns with evolving critical care paradigms emphasizing early, holistic phenotyping to guide targeted interventions (e.g., immunomodulation, nutritional optimization) and resource allocation (45). By combining acute severity indicators with vulnerability indicators, transition strategies from acute resuscitation to chronic critical care management can be informed.

Our subgroup analyses revealed critical interactions between clinical variables and biomarker profiles in modulating CCI progression risk. Notably, the heightened susceptibility observed in patients with elevated SOFA scores (*p* < 0.001) underscores the compounding effect of pre-existing organ dysfunction when coupled with high NLR/low PNI levels. This aligns with the pathophysiology of systemic inflammation impairing recovery. Similarly, vasoactive drug-dependent patients exhibited significant interaction effects (*p* < 0.001), where the Q4 group demonstrated a 5.2-fold increased risk compared to Q1. This suggests hemodynamically unstable patients experience amplified NLR/PNI-related harm: in the hyperinflammatory state, cytokines such as IL-6 and TNF-*α* are released in large quantities, which inhibit the expression and signaling of α1-adrenergic receptors, and attenuate the vasoconstrictor effect of drugs such as norepinephrine (46, 47). In addition, these cytokines also increase the activity of inducible nitric oxide synthase (iNOS), leading to vascular smooth muscle diastole and counteracting the effects of vasoconstrictive drugs (48). Moreover, albumin is the major binding protein for most vasoactive drugs (e.g., epinephrine, norepinephrine), and hypoalbuminemia also causes a decrease in protein binding and accelerated metabolic clearance of these drugs (49). While nasal nutrition showed no statistically significant interaction (*p* = 0.26), directional trends warrant discussion—the non-nutrition subgroup’s steeper risk gradient (Q4 OR = 4.62 vs. Q1) implies nutritional support might partially mitigate biomarker-associated risks, though confounding by disease severity cannot be excluded. Importantly, consistency across primary and sensitivity analyses strengthens the robustness of NLR/PNI as prognostic indicators.

Our study also has some limitations. First, in our study, the relevance and predictive value of NLR and PNI for in-hospital death in patients with CCI was not as great as for progression to CCI in critically ill patients (Tables 2, 3). This may be because the variables we selected for the study were at the level of the patient at the time of admission to the ICU, which has a limited impact on long-term prognosis. We also attempted to trace changes in the trajectories of the study variables over time. However, due to the large variations in hematologic testing intervals between individuals, we were unable to produce convincing results. Follow-up studies might be able to discuss the impact of dynamic changes in NLR and PNI on patients with CCI. Second, due to the lack of a standardized definition of CCI and the greater heterogeneity of ICU patients than the general population, our conclusions require examination in large prospective studies.

## 5 Conclusion

High NLR and low PNI levels are associated with progression to CCI and in-hospital death in critically ill patients and can be used as a valid predictive tool for poor prognosis in critically ill patients.

## Data Availability

Publicly available datasets were analyzed in this study. This data can be found at: https://mimic.physionet.org/iv/.
