# Supplementary material for: The association of neutrophil-lymphocyte ratio and prognostic nutritional index with the development to chronic critical illness and their prognosis
Source: Front Nutr. 2025 Jul 23;12:1505404. doi: 10.3389/fnut.2025.1505404 (PMC12325015; doi:10.3389/fnut.2025.1505404)
Supplement: Supplementary file 1 [file Data_Sheet_1.docx]

**Supplementary materials**

**Supplementary Table 1** Distribution of clinical factors in the definition of CCI patients

| Clinical Factors | Factor distribution | |
| --- | --- | --- |
|  | Total population (n=3661) | Study population (n=675) |
| Prolonged acute mechanical ventilation | 2952 (80.6%) | 507 (75.1%) |
| Tracheotomy | 376 (10.3%) | 32 (4.7%) |
| Stroke | 979 (26.7%) | 235 (34.8%) |
| Traumatic brain injury | 250 (6.8%) | 42 (6.2%) |
| Sepsis | 1379 (37.7%) | 287 (42.5%) |
| Severe wounds | 382 (10.4%) | 13 (1.9%) |

**Supplementary Table 2** Subgroup analyses of the association of in-hospital mortality through odds ratios for patients in the CCI group

| Subgroups | Groups | | | | *p* for interaction |
| --- | --- | --- | --- | --- | --- |
|  | Q1 | Q2 | Q3 | Q4 |  |
| Age | | | | | 0.656 |
| <65 | Ref. | 1.574 (0.549-4.780) | 2.186 (0.408-9.574) | 4.636 (1.590-14.772) |  |
| ≥65 | Ref. | 1.683 (0.746-3.851) | 2.109 (0.711-5.856) | 3.242 (1.479-7.407) |  |
| Sex | | | | | 0.240 |
| Female | Ref. | 1.372 (0.457-4.268) | 3.374 (0.918-12.019) | 5.287 (1.888-16.409) |  |
| Male | Ref. | 1.716 (0.774-3.886) | 1.211 (0.306-3.964) | 2.347 (1.056-5.388) |  |
| SOFA score | | | | | 0.479 |
| <5 | Ref. | 2.735 (0.083-52.178) | 7.631 (0.905-81.900) | 8.933 (0.176-398.601) |  |
| ≥5 | Ref. | 1.518 (0.783-3.006) | 1.497 (0.529-3.881) | 3.204 (1.684-6.306) |  |
| DM |  |  |  |  | 0.399 |
| No | Ref. | 2.177 (0.939-5.307) | 3.494 (1.200-9.909) | 4.975 (2.227-11.980) |  |
| Yes | Ref. | 1.003 (0.344-2.899) | 0.772 (0.102-3.855) | 1.894 (0.622-5.857) |  |
| COPD | | | | | 0.291 |
| No | Ref. | 1.404 (0.706-2.815) | 2.208 (0.859-5.351) | 2.517 (1.267-5.101) |  |
| Yes | Ref. | 7.193 (0.776-167.839) | 7.458 (0.200-310.372) | 24.053 (3.165-545.290) |  |
| Pneumonia | | | | | 0.253 |
| No | Ref. | 1.312 (0.321-5.999) | 0.000 (0.000-1.685*10^11^) | 1.683 (0.403-7.964) |  |
| Yes | Ref. | 1.427 (0.676-3.032) | 2.198 (0.865-5.373) | 3.609 (1.781-7.566) |  |
| Use of vasoactive drugs | | | | | 0.026 |
| No | Ref. | 2.677 (0.291-19.913) | 11.934 (2.441-66.932) | 86.993 (9.822-1151.470) |  |
| Yes | Ref. | 1.355 (0.680-2.758) | 1.044 (0.308-3.065) | 2.350 (1.212-4.707) |  |
| Nasal nutrition | | | | | 0.385 |
| No | Ref. | 12.659 (1.757-269.759) | 7.584 (0.493-219.753) | 29.659 (3.668-691.836) |  |
| Yes | Ref. | 1.291 (0.637-2.626) | 2.048 (0.767-5.088) | 2.739 (1.384-5.562) |  |

The abbreviations are as same as Table 1


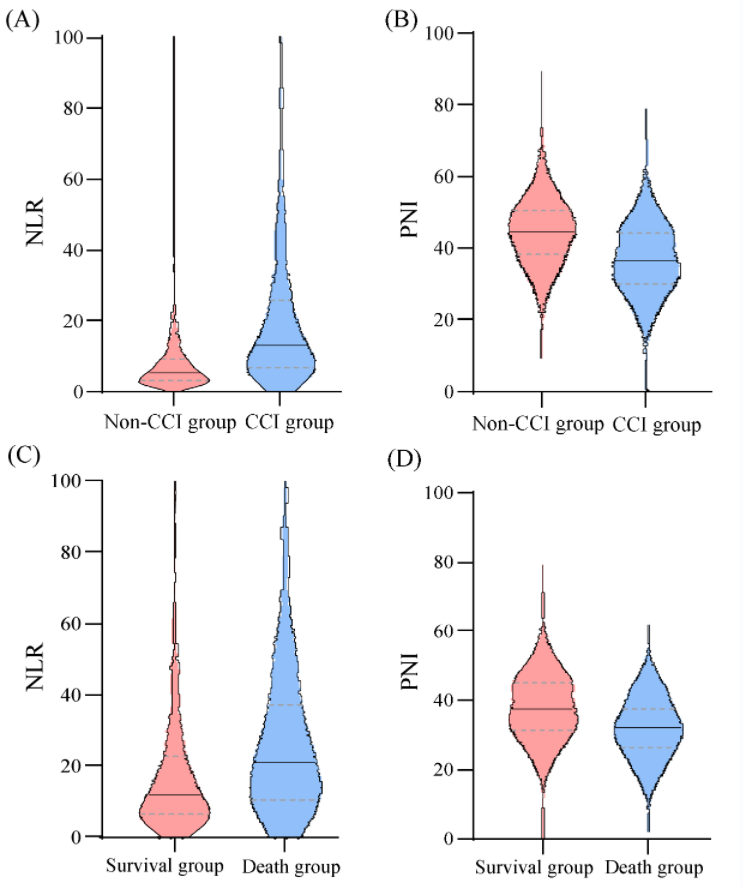


**Fig. S1** Violin diagrams for the study variables. Figures A-B, Levels of study variables in the CCI and non-CCI groups in the total population; Figures C-D Levels of study variables in the death and survival groups in the CCI cohort. In the violin figure, the solid line represents the mean, and the dotted line represents the quartile
